# Impact of Growth Hormone-Related Mutations on Mammalian Aging

**DOI:** 10.3389/fgene.2018.00586

**Published:** 2018-11-27

**Authors:** Andrzej Bartke, Nana Quainoo

**Affiliations:** ^1^Department of Internal Medicine, Southern Illinois University School of Medicine, Springfield, IL, United States; ^2^Department of Biology, University of Illinois Springfield, Springfield, IL, United States

**Keywords:** growth hormone, IGF-1, somatotropic axis, longevity genes, aging, healthspan, lifespan, dwarf mice

## Abstract

Mutations of a single gene can lead to a major increase in longevity in organisms ranging from yeast and worms to insects and mammals. Discovering these mutations (sometimes referred to as “longevity genes”) led to identification of evolutionarily conserved molecular, cellular, and organismal mechanisms of aging. Studies in mice provided evidence for the important role of growth hormone (GH) signaling in mammalian aging. Mice with mutations or gene deletions leading to GH deficiency or GH resistance have reduced body size and delayed maturation, but are healthier and more resistant to stress, age slower, and live longer than their normal (wild type) siblings. Mutations of the same genes in people can provide remarkable protection from age-related disease, but have no consistent impact on lifespan. Ongoing research indicates that genetic defects in GH signaling are linked to extension of healthspan and lifespan via a variety of interlocking mechanism, including improvements in genome and stem cell maintenance, stress resistance, glucose homeostasis, and thermogenesis, along with reductions in the mechanistic target of rapamycin (mTOR) C1 complex signaling and in chronic low grade inflammation.

## Introduction

### Aging and Longevity Reflect Impact of Environmental and Genetic Factors

Adult phenotype is determined by a complex interplay of the genetic endowment of the individual and environmental influences. Similarly to other phenotypic characteristics, the rate of aging and the longevity can be influenced by nutrition, ambient temperature, and exposure to noxious agents during development and adult life. In humans, general health, disease risk, and life expectancy are also strongly related to the availability of safe drinking water, other public hygiene factors, vaccinations, and the access to, as well as the quality of health care systems. These environmental factors influencing human health, aging, and longevity are, in turn, related to social structure and inequities between, as well as within, societies, as evidenced by a very consistent association of socio-economic status with life expectancy. Although the impact of environmental factors on aging and longevity may overshadow the effects of the genotype, the role of common and rare genetic variants and their interactions is difficult to overstate. This is perhaps best exemplified by exceptional longevity. The common perception that this characteristic “runs in the families” has been repeatedly confirmed in the studies of the offspring of centenarians and other long-lived people. These individuals are significantly healthier than their spouses, partners, or other genetically unrelated members of the same population (Adams et al., 2008; Westendorp et al., 2009; Ash et al., 2015; Gubbi et al., 2017). In addition to delayed and/or reduced incidence of various chronic diseases, familial longevity is associated with improved life expectancy and “aging well” as indicated, among others, by a more youthful appearance (Gunn et al., 2013). Additional evidence for genetic control of human aging was derived from studies of adopted children (Petersen et al., 2002) and identical versus non-identical twins (Steves et al., 2012). The complex interaction of genes and environment (“nature” and “nurture”) were recently shown to also include the impact of non-inherited maternal genetic variants on the characteristics of the child (Kong et al., 2018).

Until fairly recently, it was assumed that the genetic control of longevity, like other quantitative traits, is polygenic with numerous genes exerting relatively small, additive, synergistic, antagonistic, or protective effects. However, results obtained in the ‘1980s in a round worm, *Caenorhabditis elegans*, challenged this view by showing that mutation of a single gene can produce impressive extension of longevity (Friedman and Johnson, 1988). Since then, numerous life-extending mutations (“longevity genes” or “longevity assurance genes”) have been identified in different animal species and their actions were related to specific signaling pathways within the cells. It is the purpose of this article to review what has been learned about the endocrine control of aging in mammals from studying life extending mutations. We will focus on the impact of genes related to the somatotropic axis, which consists of the hypothalamic growth hormone releasing hormone (GHRH), the hypophyseal growth hormone (GH), and insulin-like growth factor 1 (IGF-1) on aging and longevity.

### Discovery of Longevity Genes in Worms and Insects

The discovery of longevity genes in invertebrate animals and the demonstration that mutation of a single gene can double adult lifespan and delay the appearance of many symptoms of aging in these organisms (Friedman and Johnson, 1988; Kenyon et al., 1993; Clancy et al., 2001; Tatar et al., 2001) was a major advance in the study of the genetics of aging and attracted enormous (and well deserved) attention of scientists in the field of experimental biology, as well as the general public. However, it was unclear whether these novel findings in the genetics of aging apply to more complex organisms and, in particular, to mammals. Early evidence that this may be the case (Silberberg, 1972; details in the next section of this article) appeared to have been overlooked and it was over two decades later that the extension of mammalian longevity by a single mutation was demonstrated (Brown-Borg et al., 1996).

Discoveries of mutations with major impact on longevity provided new and exciting opportunities for research. The remarkably large differences in the rate of aging between the bearers of these “longevity genes” and control (wild type) animals facilitate identification of the mechanisms of extended longevity. Search for the mechanisms responsible for slower and/or delayed aging of the long-lived mutants is further facilitated by the fact that almost all of the identified longevity genes had well known functions. This also allowed studying epistatic relationships with other genes leading to identification of signaling pathways involved in the control of aging.

Yet another advantage of using these “longevity genes” to identify mechanisms of aging is that they allow comparing long-lived and normal (control) individuals when they are young, long before their physiological characteristics are impacted by age-related changes. This is not a trivial issue because, without such genetic markers, long-lived individuals can be identified only by longer than average survival, when wild type controls for the same birth cohort are no longer available and the phenotype represents a mosaic of features related to mechanisms of extended longevity and features due to advanced age. In the case of mammals or other animals in which lifespan is measured in years rather than days (as in *C. elegans*) or weeks, availability of life extending mutations also greatly reduces the time need to study the mechanisms of aging.

### Mammalian Longevity Genes

Long before discovery of longevity genes in *C. elegans*, extension of longevity in mice homozygous for a mutation causing dwarfism was mentioned in a study of age-related osteoarthritis (Silberberg, 1972). This statement was supported by inclusion of data from dwarf mice as old as 3 years and 5 months (laboratory mice normally live two to two and a half years) but no data on average or maximal longevity of normal and dwarf mice from the employed strain was provided. These intriguing data appeared to have been overlooked by gerontologists and, curiously, a different laboratory reported in the same year that dwarf mice are extremely short-lived due to defective development and function of the immune system (Fabris et al., 1972). Incidentally, the latter observation differed from contemporary and subsequent findings in other laboratories (Shire, 1973; Schneider, 1976), and probably represented some uncommon combination of the effects of the husbandry and presence of pathogens (Shire, 1973). It was only in Brown-Borg et al. (1996) reported extension of average and maximal lifespan in both sexes of Ames dwarf mice, mutants phenotypically resembling dwarf mice studied by Silberberg (1972) and Fabris et al. (1972). Ames dwarf, Prop1^df^ mice are homozygous a for loss-of-function mutation affecting development of the anterior pituitary gland (Sornson et al., 1996) and, as a result, exhibit profound deficiency of growth hormone (GH), prolactin (PRL), and thyroid-stimulating hormone (TSH) (Bartke, 1979a,b, 2000). These endocrine defects lead to a severe decline of circulating levels of insulin-like growth factor 1 (IGF-1) and thyroid hormones, reduced growth rate, delayed maturation, and diminutive adult body size (Bartke and Brown-Borg, 2004; Bartke, 2011). Significant extension of longevity of Snell dwarf mice was reported by Flurkey et al. (2001), along with the evidence for a delay of development of various symptoms of aging. Evidence from these and more recent studies indicates that genetic deletion of GH signaling in mice slows the rate of aging (Koopman et al., 2016) and extends both lifespan and “healthspan,” the part of life free of frailty and disease (Bartke and Brown-Borg, 2004; Bartke, 2011, 2018). Studies in several laboratories demonstrated that DNA methylation changes with age and profiling of methylome can provide a surprisingly accurate “epigenetic clock” of aging (Cole et al., 2017; Wang et al., 2017). Using this approach, Cole et al. (2017) and Wang et al. (2017) provided novel evidence that aging of Snell dwarf, Ames dwarf, and GHR-/- mice is, indeed, slower than in wild type animals, resulting in a younger “biological age” in comparison to their wild type siblings.

**FIGURE 1:**
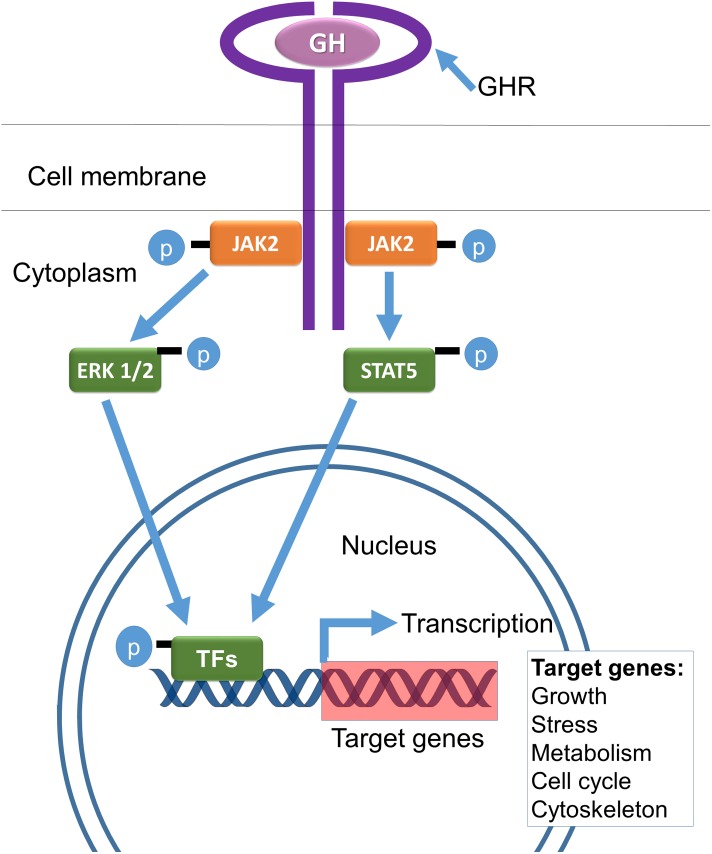
Key mechanisms of GH signaling include binding to a dimeric GH receptor (GHR) and phosphorylation (activation) of janus kinase 2 (JAK2), signal transducer and activation of transcription (STAT5), extracellular signal-regulated kinases (ERK1/2), and molecular transcription factors (TFs), leading to enhanced transcription of IGF-1 and other GH-regulated genes (adapted from Bartke et al., 2013).

Extended longevity of mice with genetic deletion of GH receptors and resulting GH resistance (Coschigano et al., 2003) and in GHRH-/- mice with deletion of hypothalamic GH-releasing hormone leading to isolated GH deficiency (Sun et al., 2013) provided evidence that suppression of GH signaling is sufficient to extend longevity of mice. Comparisons of data on longevity, mortality rate, and various measures of healthspan in these mutants indicate that slower and/or delayed aging of hypopituitary Ames dwarf and Snell dwarf mice is due primarily to GH deficiency with hypothyroidism and PRL deficiency playing only a minor, if any, role. Additional information on these and other GH signaling-related life-extending mutations in mice and humans is provided later in this article. Mechanisms of GH signaling within target cells are shown schematically in Figure 1.

### Biological Mechanisms of Aging Have Been Conserved in the Course of Evolution

Very soon after demonstration that mutation causing GH deficiency extends longevity in mice, the Ruvkun laboratory reported that genes with major impact on aging in a worm, *C. elegans*, exhibit extensive homologies to genes coding the structure of mammalian insulin and IGF-1 receptors and to genes involved in insulin/IGF-1 signaling (IIS) (Kimura et al., 1997; Tissenbaum and Ruvkun, 1998). Since IGF-1 is a key mediator of GH actions, and GH modulates both the secretion of insulin and the tissue responses to this hormone, these exciting observations linked the actions of key longevity genes in *C. elegans* with the effects of GH-related genes in mice. What emerged from these findings and from the subsequent work is the realization that an important mechanism of biological aging is evolutionarily conserved in organisms raging from yeast to mammals (Longo and Finch, 2003; Tatar et al., 2003; McElwee et al., 2007). This unexpected fact is truly astounding considering the range of differences in the structure, complexity, life history, physiological functions, and life expectancy between unicellular yeast, microscopic roundworms, insects, and mammals.

It should be emphasized that the homologies of genes and signaling pathways that control aging in diverse organisms do not imply that the mechanisms of this control are identical. Insulin/insulin-like growth factor signaling (IIS) impacts aging in worms, insects, and vertebrates. However, in vertebrates, biosynthesis of IGF-1, its circulating levels, and bioavailability, are under control of GH, which has no known homologs in invertebrates. The interaction between GH and IIS in the control of aging is further complicated by complex physiological relationships among GH, IGF-1, and insulin. Thus, many of the actions of GH are mediated by IGF-1, but IGF-1 also exerts actions which are different and, in some cases, opposite to the actions of GH. For example, GH promotes lipolysis, while IGF-1 enhances the sensitivity of adipose tissue to insulin, leading to fat deposition. Moreover, GH promotes insulin secretion, but opposes its actions by inducing insulin resistance. In humans, divergent effects of IGF-1 on the risks of different age-related diseases add yet another element to this complexity. Epidemiological studies indicate that low levels of serum IGF-1 protect from cancer but increase the risk of cardiovascular disease.

Discovery of this highly conserved genetic network that controls aging in evolutionarily distant organisms, served to focus attention on the tradeoffs between various physiological process and on the reciprocal relationships among aging and anabolic processes, growth, and maturation. These relationships are currently investigated in many laboratories, also including our group. Growth-related and anabolic processes stimulated by GH, IGF-1, insulin, and mTORC1 signaling apparently have definite “costs” in terms of longevity. As was already mentioned earlier in this article, genetic, nutritional, or pharmacological suppression of these signaling pathways in mice produce major extension of longevity (Harrison et al., 2009; Bartke, 2011, 2018). A recent study indicates that differences in cell size, an mTOR-dependent trait, are correlated with lifespan differences among 24 mammalian species (Anzi et al., 2018).

### Growth Hormone-Related Mutations With Major Impact on Mouse Longevity

In this section, we will survey mutations and targeted gene deletions (“knock-outs”) which block or severely diminish somatotropic signaling in mice and produce significant extension of longevity. Effects on these longevity genes in mice will be compared to the effects of the corresponding mutations in humans on age-related disease and longevity.

#### Genetic Defects in Development of the Adenohypophysis Leading to Deficiency of GH and Other Hormones

Hypopituitarism has been reported in Snell dwarf (*Pit1*^dw/dw^) mice due to a loss-of-function mutation in the pituitary-specific factor 1 (*Pit1*) gene, also known as POU domain, class 1, transcription factor 1 (*Pou1f1*) gene, which is involved in the differentiation of a specific cell lineage of the anterior pituitary during ontogenesis. The resulting lack of somatotroph, thyrotroph, and lactotroph cells in this gland (Bartke, 1979a; Li et al., 1990) leads to endocrine abnormalities, including the reduction of the circulating levels of growth hormone (GH), insulin-like growth factor 1 (IGF-1), thyroid stimulating hormone (TSH), thyroid hormones (T3 and T4) and prolactin (PRL) (Bartke, 1979a,a, Li et al., 1990). These endocrine deficits are associated with a major extension in lifespan (Flurkey et al., 2001). The extension of longevity has been also observed in Ames dwarf (*Prop1*^df/df^) mice, which have the same endocrine abnormalities as Snell dwarf mice, but due to a recessive mutation in another gene, the Prophet of Pit1 (*Prop1*) gene, also involved in the differentiation of somatotrophs, thyrotrophs, and lactotrophs (Brown-Borg et al., 1996; Sornson et al., 1996; Bartke and Brown-Borg, 2004). However, there is no evidence for longevity extension in humans with mutations in the same genes (Krzisnik et al., 2010).

The expression of *Prop1* gene precedes the expression of *Pit1* gene, whose transactivation leads to the development of somatotroph, thyrotroph, and lactotroph cells in the anterior pituitary, also the expression of genes that code for GH, TSH beta subunit, and PRL hormones (Li et al., 1990; Sornson et al., 1996). Mutations in *Pit1* gene were first observed in Snell and Jackson dwarf mice (Li et al., 1990; Flurkey et al., 2001) and cause combined pituitary hormone deficiency (CPHD), and hypoplasia of the anterior pituitary in both mice and humans (Li et al., 1990; Pernasetti et al., 1998; Nakamura et al., 1999; Vallette-Kasic et al., 2001; Cohen and Radovick, 2002; Hashimoto et al., 2003; Kelberman et al., 2009). In humans, a point mutation in the *PIT1* (*POU1F1*) coding sequence has been found in a number of CPHD patients, additionally, there is evidence that the DNA-binding capacity of the PIT1 protein is impacted in this condition (Ohta et al., 1992; Cohen et al., 1995; Pernasetti et al., 1998). Defects in transcription factor genes such as *PROP1/Prop1*, *POU1F1*/*Pou1f1*, HES homeobox 1 (*HESX1*/*Hesx1*), LIM homeobox 3 (*LHX3*/*Lhx3*) and LIM homeobox 4 (*LHX4*/*Lhx4*) have all been known to cause CPHD in humans and mice (Wu et al., 1998; Bertko et al., 2017). Deletion of *PROP1* gene yields abnormally short proteins that fail at regulating other genes, resulting in significant impairment of pituitary cell differentiation (Fang et al., 2016). While dwarf phenotype in Ames dwarf mice is expressed only in animals that are homozygous for *Prop1* gene mutation, most *PROP1*-related phenotypes in humans are observed in both homozygous and compound heterozygous patients (Lap-Yin and Wai-Yee, 2010). The mutant PROP1 protein in humans has its DNA-binding homeodomain affected, leading to a severely reduced DNA binding and/or gene transactivation activity of the transcription factor as compared to the PROP1 mutant protein found in Ames dwarf mice (Lap-Yin and Wai-Yee, 2010; Bertko et al., 2017). It is worthy to note, while hypopituitary *Prop1*^df/df^ mice live longer then their normal siblings, hypopituitarism in humans was reported to be a risk factor for cardiovascular diseases. However, *PROP1* gene mutation studies performed in patients with dwarfism from the Krk Island in the Adriatic Sea have shown that these patients can live as long, or perhaps even longer, than normal subjects in the same population.

#### Deletion and Mutation of the GH Receptor Gene Leading to GH Resistance

In addition to the effects of the aforementioned naturally occurring gene mutations, dwarfism and extended lifespan can also result from targeted disruption of GH receptor (*Ghr*) gene in GHR knockout (GHR-/-) mice, a model for human’s Laron syndrome (Coschigano et al., 2003). Recent report by List et al. (2011) critically examined the parallels between GHR-/- mice and humans with GH insensitivity, and observed a striking similarity in phenotypic traits. Provided that suppression of GHR signaling in GHR-/- mice leads to major extension of longevity, could the same be concluded about patients with Laron syndrome?

The inability of receptors to bind GH and their failure in inducing GH signaling in cells, caused by the mutations seen in Laron syndrome patients, has been related to protection from age-related disease, as reported by Shevah and Laron (2007) and by Guevara-Aguirre et al. (2011) in a study of Ecuadorian subjects. Guevara-Aguirre et al. (2011) monitored for 22 years a group of subjects carrying mutations in the *GHR* gene and collected information on the effects of absent GH signaling, including surveys on causes and ages of death in this cohort. The authors provided clear evidence that the Laron syndrome patients had significantly lower risks of developing cancer and diabetes but their longevity was not significantly altered. Studies on the effects of GHR dysfunction on longevity in humans are complicated due to the genetic architecture of human longevity and the numerous factors that are associated with survival at various ages (Christensen et al., 2006). These patients presented diminished levels of circulating IGF-1, a hormone that plays an important role in cancer. In this study, the majority of GHR-deficient Laron syndrome patients were homozygous for an A to G splice site mutation at position 180 in exon 6 of the *GHR* gene. In an *in vitro* study performed by these authors, human mammary epithelial cells (HMECs) were incubated in medium supplemented with serum from either GHR-deficient (GHRD) subjects or their normal relatives. Results showed that the cells incubated with serum of GHRD patients presented fewer DNA breaks, but higher apoptosis rate, and reduction in expression of several genes in a fashion that promotes cellular protection and lifespan extension. An increase in DNA damages was observed when IGF-1 was added to the serum from Laron syndrome patients, providing evidence that the deficiency of circulating IGF-1 levels in GHRD patients is the major reason for resistance to oxidative stress in those subjects (Guevara-Aguirre et al., 2011). Nevertheless, even though this study provided compelling evidence that Laron syndrome patients are protected from age-related pathologies, surprisingly, no lifespan extension was observed in this group. This could have been possibly due to different causes of death: a major proportion (70%) consisted of convulsive disorders, alcohol toxicity, accidents, liver cirrhosis, and other non-age-related causes (Guevara-Aguirre et al., 2011). Additionally, the GHRD patients also presented a lack of type 2 diabetes, despite the obese phenotype typically displayed in this group. This is intriguing, and has been related to decreased insulin levels and a lower insulin resistance in GHRD subjects. In *Ghr-/-* mice with GHRD, increased insulin sensitivity is believed to represent an important mechanism of their remarkable extension of longevity (Bonkowski et al., 2006). Taken together, the available data indicate that GHR dysfunction in Laron syndrome patients protects them from cancer and diabetes, but has no significant impact on longevity.

#### Isolated GH Deficiency Resulting From GHRH or GHRH-R Mutations

Sun et al. (2013) reported lifespan extension in growth hormone releasing-hormone (GHRH) knockout mice (Alba and Salvatori, 2004a), which was comparable to those observed in Snell dwarf^dw/dw^ and Ames dwarf^df/df^, and GHR-/- mice. Calorie restriction (CR) produced further extension of longevity in GHRH-/- mice, differing from the effects of CR in GHR-/- animals (Bonkowski et al., 2006). These results suggest additive effects of GHRH mutation and CR, and imply that the mechanisms of their effects on longevity are not identical, although undoubtedly overlapping. Interestingly, no *GHRH* gene mutations have been reported in humans, as opposed to GHRH receptor (*GHRHR*) gene mutations, which have emerged as a common cause of inherited autosomal recessive isolated GH deficiency (IGHD) also known as Dwarfism of Sindh (a province in Pakistan) (Alba and Salvatori, 2004b; Aguiar-Oliveira et al., 2010). The IGHD in mice and humans, caused by GHRHR gene defects, highlights the important role of this receptor in the regulation of GH synthesis and secretion, as well as aging and longevity (Flurkey et al., 2001; Aguiar-Oliveira et al., 2010). Mutations of the human *GHRHR* were discovered in families with consanguineous marriages (Alba and Salvatori, 2004b). Subjects homozygous for these mutations have short stature, proportional dwarfism, apparent extension of healthspan, but no extension of longevity (Aguiar-Oliveira et al., 2010). This is based on evidence from over 25 years of studies of Aguiar-Oliveira and his colleagues in a large kindred of IGHD subjects who did not receive GH replacement therapy (Aguiar-Oliveira et al., 2010).

### Reduced IGF-1 Signaling Influences Aging

Insulin-like growth factor 1 (IGF-1) represents an important mediator of GH actions, and both molecules play important roles in aging and longevity (Bartke, 2017). Mice heterozygous for deletion of the IGF-1 receptor (IGF1R) gene are long-lived, although the magnitude of life extension depends on sex and genetic background (Xu et al., 2014). The beneficial impact of reduced IGF-1 signaling on longevity is pronounced in females, but very small or absent in males (Xu et al., 2014). Studies in *Igf1r^+/-^* mice and in mice with deletion of *Igf1r* gene in different cell types provided evidence for unexpected neuroprotective effects of reduced IGF-1 signaling and, thus, greatly expanded the present understanding of the role of IGF-1 in the central nervous system (Cohen and Dillin, 2008; Chaker et al., 2015). Blockade of IGF1 receptors enhanced and their activation prevented hypothermia in mice exposed to CR (Cintron-Colon et al., 2017). These intriguing observations link brain IGF-1 signaling to nutrients and body temperature homeostasis, major regulators of metabolism and aging.

Mice with reduced levels of IGF-1, due to homozygous insertion of the IGF-1 gene, have increased maximal lifespan, although mean lifespan was not consistently affected (Lorenzini et al., 2014). These animals are smaller than wild type controls and exhibit resistance to detrimental effects of high fat diet (Salmon et al., 2015).

Studies in humans have associated the decreased levels of IGF-1 to extended longevity and chances to become a centenarian (Milman et al., 2014). Bonafè and collaborators reported an association between insulin/IGF-1 pathway and longevity in a group of long-lived people in Italy (Bonafe et al., 2003). Results from a study of Ashkenazi Jewish centenarians in the United States, also linked *IGF1R* gene polymorphisms with extreme longevity (Suh et al., 2008).

The bioavailability and actions of IGF-1 are regulated by its interactions with IGF-binding proteins (IGFBP), carrier proteins that may enhance or inhibit IGF signaling. The levels of IGFBPs are influenced by IGFBP proteinases (Conover and Bale, 2007; Conover et al., 2010). Genetic deletion of pregnancy-associated plasma protein-A (PAPPA), a zinc metalloproteinase that enhances IGF signaling through cleavage of inhibitory IGFBP has been found to extend maximum lifespan in mice (Conover and Bale, 2007; Conover et al., 2010). Reduced *Pappa* activity increases the levels of IGFBPs, which results in reduced IGF signaling and leads to increased longevity (Conover and Bale, 2007; Conover et al., 2010). Conover and colleagues reported a significant increase of lifespan in both sexes of PAPPA-/- mice in contrast to findings in mice with reduced IGF-1 or IGF-1 receptor levels (Lorenzini et al., 2014; Xu et al., 2014). It is however important to note that the extended longevity phenotype found in PAPPA-/- mice is associated with normal serum levels of IGF-1(Conover and Bale, 2007). Conover and Bale (2007) suggested that the control of the availability of IGF-1 at local, tissue level and the moderate reduction rather than the complete inhibition of IGF-1 signaling is the key to lifespan extension. Survival data collected in the PAPPA-/- mice study showed that the knockouts experienced a reduction in age-related degenerative diseases and displayed a lifespan extension of about 30%, with degenerative diseases being the cause of high mortality in wild type mice (Conover and Bale, 2007).

Mechanisms linking effects of deleting or silencing GH-related genes to the extension of healthspan and lifespan will be discussed in the next section of this article. However, before addressing this subject, we would like to indicate that longevity of laboratory mice can also be affected by altering the expression of genes not related to IIS or GH signaling. A partial list of these genes is provided in Table 1. Although for some of these genes, the changes in longevity are relatively small, limited to only one sex, or concerning only average or median (rather than maximal) longevity, much has been and, undoubtedly, will continue to be learned from the study of their actions.

**Table 1:** Mouse longevity genes not directly related to GH signaling.

Gene	Genetic modification that extends life	Reference
Insulin receptor (in adipose tissue; FIRKO)	KO	Bluher et al., 2003
Insulin receptor substrate 1 (*Irs1*)	KO	Selman et al., 2008
Insulin receptor substrate 2 (*Irs2*)	KO	Taguchi et al., 2007
S6K1 protein kinase	KO	Selman et al., 2009
Catalase (mitochondrial over expression)	Tg	Schriner et al., 2005
Klotho (transgenic overexpression)	Tg	Kurosu et al., 2005
Adenylyl cyclase type 5 (AC5)	KO	Vatner et al., 2015
Insulin (*Ins1^-/-^ Ins2^+^*^/-^)	KO	Templeman et al., 2017
Regulator of G protein signaling 14 *Rgs14*	KO	Vatner et al., 2018
Beclin J-BCL2 complex	Mutation knock-in	Fernández et al., 2018

*KO, targeted disruption (“knockout” KO) of the gene; Tg, transgenic expression*.

### Multiple Mechanisms Are Linking GH-Related Genes to Aging

Much of the work in our laboratory during the last 30 years was directed at identifying mechanisms of extended longevity of mice with GH-related mutations and answering the question how major reduction or absence of normal endocrine signals can have major beneficial impact on healthspan and lifespan. These issues have also been addressed by others with major contributions from the Kopchick, Brown-Borg, Miller, Papaconstantinou, and Masternak laboratories (List et al., 2011; Brown-Borg, 2015; Basu et al., 2018; Masternak et al., 2018).

Both GH-deficient and GH-resistant mice have many phenotypic characteristics that presumably account for, or contribute to, healthy aging and extended longevity and, thus, represent likely mechanisms of these effects. These characteristics include increased resistance to multiple stressors such as free radicals and toxins (Brown-Borg, 2006; Bokov et al., 2007), reduced chronic low grade inflammation, senescent cell burden, and expression of pro-inflammatory cytokines in the central nervous system (Kirkland et al., 2010; Hascup et al., 2015; Spadaro et al., 2016), reduced mTORC1 and increased mTORC2 signaling (Sharp and Bartke, 2005; Dominick et al., 2015; Fang et al., 2018), as well multiple adaptations of carbohydrate, lipid, and energy metabolism (Brown-Borg and Bartke, 2012; Bartke, 2017; Bartke, 2018). Many of the physiological characteristics of GH-related mutants interact, forming a complex network of mechanisms. For example, reductions in the levels of pro-inflammatory cytokines, the number of senescent cells, the secretory capacity of pancreatic beta cells, and mTORC1 signaling, interact with increased levels of adiponectin and reduced GH signaling to improve insulin sensitivity, while each of these factors also influences aging by other mechanisms (Tatar et al., 2003; Bartke and Brown-Borg, 2004; Brown-Borg and Bartke, 2012; Bartke, 2018). We believe that the remarkable extension of longevity in mice with genetic GH deficiency or resistance results from alterations in multiple mechanisms of aging and interactions among these alterations. While complete listing and detailed discussion of mechanisms linking reduced GH signaling with the extension of healthspan and lifespan is outside the scope of this article, tradeoffs between reproduction and longevity, and the role of stress resistance, will be outlined below.

Somatic growth regulated by GH and IGF-1 is linked to sexual maturation, which is delayed in GH deficient and GH resistant organisms (Bartke et al., 2013; Hoeflich et al., 2016). Though fertile, both female and male hypopituitary and GHR-/- mice experienced a significant delay in puberty, which leads to a reduction in fecundity and a lower reproductive fitness (Bartke and Brown-Borg, 2004; Bartke et al., 2013). Sexual maturation in male and female GHR-/- mice is delayed by approximately 1 week, and humans with Laron dwarfism were reported to exhibit a similar reproductive phenotype (Bartke et al., 2013). Extended longevity has been associated with reduced fecundity, as decreased reproduction leads to a diminished use of resources allocated to the maintenance of somatic tissues (Mukhopadhyay and Tissenbaum, 2007). Extensive studies have been performed in order to investigate the effect of reproduction on longevity both in humans and lower life forms (Doblhammer, 2000), but varying results have been reported. Many clinical studies have shown a positive relationship between longevity and reproduction (Doblhammer, 2000; Muller et al., 2002; McArdle et al., 2006), while others have suggested a trade-off or no association between these two factors (Voland and Engel, 1986; Kirkwood and Rose, 1991; Le Bourg et al., 1993; Korpelainen, 2000; Smith et al., 2009; Tabatabaie et al., 2011). The depletion of resources necessary for self-maintenance in an attempt to be used for reproductive purposes has been associated with decreased longevity. To gain further insights into the relationship between longevity and reproduction, Tabatabaie et al. (2011) studied two unrelated groups of exceptionally long-lived Ashkenazi Jewish individuals, and observed a trade-off between longevity and reproduction. Evidence that reduced reproduction is, in fact, associated with longevity is, however, debatable, and this topic continues to generate controversies (Westendorp and Kirkwood, 1998; Le Bourg, 2007). Physiological cost of reproduction in females is much higher than in males, and this further complicates interpretation of the available data (Voland and Engel, 1986; Le Bourg et al., 1993).

Additional factors that affect aging and longevity are environmental and cytotoxic stresses. Oxidative stress is believed to be a major cause of aging in a wide range of laboratory model organisms, and numerous studies have shown that animals carrying mutations which confer resistance to oxidative stress are long-lived (for some examples: Sun and Tower, 1999; Finkel and Holbrook, 2000; Bartke et al., 2001; Holzenberger et al., 2003). Recent studies on the genetics of aging using fruit fly *Drosophila melanogaster*, yeast *Saccharomyces cerevisiae*, nematode Caenorhabditis elegans, and ocean quahog *Arctica islandica*, provided compelling evidence that enhanced resistance to stress is associated with extended longevity (Jazwinski, 2000; Johnson et al., 2002; Salmon et al., 2005; Ungvari et al., 2011). Murakami and colleagues investigated resistance to multiplex stresses in cultures of tail skin cells from young Snell dwarf (*Pit1*^dw/dw^), and wild type (control) mice using a variety of potentially lethal stresses, ultraviolet light, heat, paraquat, hydrogen peroxide, and the toxic metal cadmium. Results demonstrated that the cells derived from dwarf mice were more resistant to noxious agents (Murakami et al., 2003). The study was subsequently extended to show that dermal fibroblast cells derived from young adult long-lived Ames dwarf^df/df^ and GHR-/- mice are also resistant, *in vitro*, to the cytotoxic effects of hydrogen peroxide, cadmium, ultraviolet light, paraquat, and heat (Murakami et al., 2003; Salmon et al., 2005; Bartke, 2012).

### Growth Hormone and Genes Related to GH Signaling Are Involved in the Control of Human Aging

The negative association of somatotropic (GH/IGF-1) signaling with longevity discovered in laboratory rodents applies to other mammalian species, apparently including humans. Thus, exceptional longevity in humans has been associated with reduced IGF-1 levels (Milman et al., 2014) and insulin/IGF-1 composite signaling score (van Heemst et al., 2005). In many populations, shorter people live longer (Samaras, 2007; He et al., 2014), and offspring of long-lived families secrete less GH than their spouses (van der Spoel et al., 2016). Moreover, mortality is increased in individuals with pathologic excess of GH (Baumann, 1987). Studies of the genetic polymorphism of candidate genes, genome-wide association studies (GWAs), and analysis of signaling pathways, genetic networks, and copy number variations, provided evidence that human aging is influenced by genes related to the somatotropic axis (van Heemst et al., 2005; Suh et al., 2008; Bonafe and Olivieri, 2009; Dato et al., 2018) and its downstream targets including FOXO3A (Blankenburg et al., 2018; Revelas et al., 2018; Zhao et al., 2018).

Surprisingly, mutations leading to profound suppression of GH secretion or to GH resistance have no major or consistent effect on human longevity (Aguiar-Oliveira et al., 2010; Krzisnik et al., 2010; Guevara-Aguirre et al., 2011), as was mentioned earlier in this article. However, they can provide protection from aging-related diseases including cancer, diabetes, and atherosclerosis (Aguiar-Oliveira et al., 2010; Guevara-Aguirre et al., 2011), in spite of changes in body composition and serum lipids that could be described as unfavorable. There is also intriguing evidence that severe genetic GH deficiency can reduce age-related changes in muscle function, cognition, and behavior leading to extension of the healthspan and “healthy aging” (Aguiar-Oliveira et al., 2017; Nashiro et al., 2017).

The fact that mutations leading to severe suppression or absence of GH signals have a major beneficial impact on longevity in mice but not in people is, perhaps, not surprising. There is increasing evidence for tradeoffs between growth, and anabolic processes that favor sexual maturation and fecundity, and the maintenance and repair mechanisms that promote longevity. Mice represent one of the extreme examples of short-living species that “live fast,” mature early, and can produce many offspring, but die young, while humans have roughly the opposite characteristics and are extremely long-lived in comparison to other mammals. Thus, suppression of the expression of growth-related genes and reduced GH action would have a much greater impact on aging in mice than in people. The fact that stress resistance and DNA repair are enhanced in mice with life extending GH-related mutations (Murakami et al., 2003; Bokov et al., 2004; Salmon et al., 2005; Podlutsky et al., 2017) would seem to support this reasoning. Although less likely, phenotypic differences between humans and mice or between different cohorts of subjects with hereditary disruption of GH signaling could also be related to differences in the exact nature of underlying genetic defects. For example, GH resistance of “Laron dwarf” GHR-/- mice is due to deletion of most of the fourth exon and part of the fourth intron of the Ghr gene (Coschigano et al., 2003), while Laron syndrome in different human cohorts is due to various deletions, splice variants, nonsense, missense, or frameshift mutations (Berg et al., 1993; Janecka et al., 2016).

## Author Contributions

AB: article conception and design, data acquisition, data analysis and interpretation, receiving grant, and approval of final manuscript. AB and NQ: wrote the manuscript.

## Conflict of Interest Statement

The authors declare that the research was conducted in the absence of any commercial or financial relationships that could be construed as a potential conflict of interest.
